# Magnesium oxide tablets form rigid masses in xanthan gum-based thickeners: A physicochemical hazard revealed by a clinical case

**DOI:** 10.1371/journal.pone.0361494

**Published:** 2026-10-29

**Authors:** Soichi Makino, Miyu Ooe, Kazunari Miyazawa, Shogo Kayano, Sayaka Kominato, Yoshinori Saito, Ryohei Hirota, Yuta Watanabe, Fumiko Hayano

**Affiliations:** 1 Department of Radiology, Shin-Kuki General Hospital, Saitama, Japan; 2 Department of Radiology, University of Occupational and Environmental Health, Fukuoka, Japan; 3 Department of Radiology, Showa General Hospital, Tokyo, Japan; 4 Department of Pharmacy, Shin-Kuki General Hospital, Saitama, Japan; 5 Department of Clinical Laboratory, Shin-Kuki General Hospital, Saitama, Japan; 6 Department of Rehabilitation, Shin-Kuki General Hospital, Saitama, Japan; 7 Department of Breast Surgery, Shin-Kuki General Hospital, Saitama, Japan; National University of Rosario, ARGENTINA

## Abstract

Dysphagia, or difficulty swallowing, is a matter of serious concern among older adults. While thickening agents are essential for dysphagia management, they are not pharmacologically inert; their physicochemical interactions with oral medications remain poorly understood and can pose an iatrogenic hazard of mechanical esophageal obstruction. Following a case of acute esophageal obstruction of unknown origin, this study aimed to identify the foreign body, to assess the reproducibility of any disintegration failures across formulations and thickeners, and to examine the systemic barriers that would allow such a hazard to remain unaddressed. This foreign body, a magnesium oxide (MgO) tablet, caused obstruction in a malignant esophageal stricture. It was analyzed using *ex vivo* computed tomography and chemical analysis. A static immersion model was used to mimic esophageal stasis. The disintegration behaviors of four MgO formulations (n = 6 each) were compared using thickeners (1.0–3.0%) and a pharmaceutical swallowing aid jelly (SA-J). Furthermore, a cross-sectional market survey was conducted in nine regions in Japan to assess the economic barriers to the clinical adoption of SA-J as an alternative swallowing aid. The foreign body was identified as a non-disintegrated, swollen MgO mass. *In vitro* experiments confirmed a consistent phenomenon observed across all tested formulations: thickeners completely inhibited disintegration in every combination tested, increasing tablet weight by approximately 2.2-fold at concentrations of 2.0–3.0% and forming rigid masses. Conversely, the tablets disintegrated in SA-J in approximately 25 s. The market survey revealed that SA-J costs were approximately 14.5-fold higher per administration than thickeners (p < 0.001). Although based on a single case, these findings highlight that xanthan gum-based thickened water represents a physicochemical hazard that may remain unrecognized in current clinical practice. Choice of swallowing vehicle should be made with full awareness that xanthan gum-based thickeners can prevent the disintegration of MgO tablets.

## Introduction

Dysphagia, or difficulty swallowing, poses a growing clinical burden in aging societies worldwide. In Japan, aspiration pneumonia — a well-recognized consequence of dysphagia — accounts for nearly 50,000 deaths annually, reflecting the country’s rapidly aging demographic in which approximately 30% of the population is aged 65 years or older [1,2]. To prevent aspiration pneumonia, thickening agents are used widely to increase liquid viscosity [3]. In nursing and dietary care, these thickeners are used not only with meals, but also with oral medications in polypharmacy patients [4]. Yet thickening agents and oral medications, while individually safe, may introduce an iatrogenic hazard in combination.

Thickeners are commonly employed for preventing aspiration, but they are not inert. They alter the rheology and water availability of liquids, potentially affecting drug disintegration and dissolution. Current pharmacopeial standards, including the Japanese Pharmacopoeia (JP) and the United States Pharmacopeia, mandate dissolution tests in water or buffer solutions; however, these tests do not account for the complex real-world environments created by additives [5]. Consequently, tablet behavior during dysphagia management remains insufficiently characterized. Among oral medications, magnesium oxide (MgO), a first-line osmotic laxative, warrants particular attention for two reasons: it is widely prescribed in Japan owing to its well-established efficacy and safety [6], and its disintegration depends on hydration, rendering it susceptible to the water-binding properties of thickeners. Despite the widespread clinical practice of administering oral medications with thickened water, the physicochemical compatibility of this combination has not been systematically evaluated.

The present study was prompted by a clinical event involving an 81-year-old man who was referred for radiation therapy for advanced esophageal cancer (cT4) with a malignant stricture. A JP-compliant MgO tablet, administered with xanthan gum-based thickened water, failed to disintegrate and remained lodged in the middle of the esophagus for over 70 h. Imaging confirmed that the residue retained the original morphology of the tablet, which necessitated endoscopic removal using a basket catheter. This observation suggested that thickening agents may inhibit the disintegration of MgO tablets, causing them to swell after absorbing moisture and thus increasing the physical hazard of mechanical obstruction, particularly in patients with impaired gastrointestinal passage. We addressed three sequential objectives. First, to identify the nature of the foreign body retrieved from the index case. Second, based on the findings of this identification, we aimed to determine using systematic *in vitro* characterization whether this incident represented an isolated event or a reproducible phenomenon. This included examining the influence of thickener concentration, thickener brand, and MgO formulation on disintegration behavior, and evaluating whether safer swallowing vehicles exist. Finally, we sought to examine the structural barriers, including economic factors, that prevent this hazard from being recognized and addressed in clinical practice.

## Materials and methods

### Ethical considerations

This study was approved by the Institutional Review Board (IRB) of Shin-Kuki General Hospital (Approval No. 121 and 134) and was conducted in accordance with the principles of the Declaration of Helsinki. Written informed consent for publishing the clinical case details and associated images was obtained from the patients. The clinical cases included in this study were identified between 19/08/2025 and 08/04/2026. The nurse interviews and the market price survey of swallowing aid vehicles were submitted to the IRB and formally determined to be exempt from full review. This exemption was granted because the interviews were part of routine clinical quality improvement with no identifiable personal information collected, and publicly available retail data was only used. Medical records and patient-derived research data were accessed for research purposes from 28/09/2025–08/04/2026, in accordance with the aforementioned IRB approval. The authors had access to information that could identify individual participants during data collection, as this study involved individual clinical cases; however, all identifiable information was anonymized prior to publication.

### Identification of the extracted object

#### Sample pretreatment and definition.

Upon retrieval, the foreign body was found to be encapsulated in a colorless gelatinous film. Although this film was not independently analyzed at the time of retrieval, it was later identified as residual xanthan gum-based thickener material based on the findings of *in vitro* experiments and clinical history. This film was manually removed, and the remaining solid core was designated “Object X.” All subsequent analyses were performed on Object X.

#### Radiological analysis via computed tomography (CT).

*Ex vivo* CT analysis was performed to identify Object X. Based on the medication history of the patient, three candidates were selected *a priori*: betamethasone (0.5 mg), famotidine (10 mg), and MgO (F-O: 330 mg; Magnesium Oxide Tablets 330 mg “YOSHIDA”, Yoshida Pharmaceutical Co., Ltd., Tokyo, Japan). The candidates and Object X were scanned using the same scanner and protocol as the clinical CT scan of the patient (Aquilion Serve; Canon Medical Systems, Tochigi, Japan). The mean CT attenuation (Hounsfield units [HU]) of each candidate was measured using standardized regions of interest. The most likely identity was inferred by comparing the CT attenuation values, morphology, and internal structure.

#### Physicochemical verification.

To exclude the possibility of a dispensing error, Object X was physically characterized. The weight of Object X was measured using a standard laboratory balance (precision: 0.01 g; Ishida Co., Ltd., Kyoto, Japan), and its dimensions were measured using calipers. The core was fractured to examine its internal structure.

#### Chemical analysis.

As an additional verification step, approximately half of Object X (250 mg) was ground with a mortar and pestle and suspended in 500 mL of distilled water. For comparison, half of a control F-O (207 mg) was suspended under identical conditions. Because Object X was hypothesized to be a swollen MgO tablet derived from a single 330 mg tablet, half of each sample was used to enable direct qualitative comparison. Each suspension was centrifuged at 1,700 × *g* for 5 min (H-19α; Kokusan, Saitama, Japan). The supernatant was collected, and Mg was quantified using an enzymatic method [7] with an automated biochemical analyzer (measurable range: 0.1–20.0 mg/dL; TBA-nx360; Canon Medical Systems, Tochigi, Japan).

Alkalinity was evaluated qualitatively through a colorimetric reaction using levodopa, which is known to degrade and darken under alkaline conditions [8]. Briefly, one-quarter of each sample—Object X (125 mg), F-O (100 mg, equivalent to one-quarter of a tablet), and a levodopa tablet (50 mg) as a positive control—was pulverized and suspended in 50 mL of distilled water. Each suspension was then combined with a levodopa solution (50 mg in 50 mL of distilled water) to obtain a final volume of 100 mL. After 1 h of incubation, the color change was visually inspected. These biochemical tests, including Mg concentration measurement and colorimetric reactions, were conducted in the clinical laboratory of Shin-Kuki General Hospital, where conditions were consistently maintained (temperature range: 23–25 °C; relative humidity: 39%–44%).

### Study for elucidating the in vitro mechanism

#### Nurse interviews.

To establish clinically valid experimental conditions, interviews were conducted with all 10 nurses who were directly responsible for managing patient medication. The interviews revealed that six nurses prepared thickeners at a concentration of 1.0%, whereas four used 3.0%. This variation guided the choice of an experimental concentration range of 1.0% to 3.0%.

#### Weight uniformity verification.

Four commercially available JP-compliant MgO 330 mg formulations were tested: F-O (previously described); F-1, Magmit Tablets 330 mg (Shioe Pharmaceutical Co., Ltd., Hyogo, Japan/ Magmitt Pharmaceutical Co., Ltd., Kagawa, Japan); F-2, Magnesium Oxide Tablets 330 mg “MOCHIDA” (Mochida Pharmaceutical Sales Co., Ltd., Tokyo, Japan); and F-3, Magnesium Oxide Tablets 330 mg “KENEI” (Kenei Pharmaceutical Co., Ltd., Osaka, Japan). To assess baseline variability among formulations, the dry weights of all four formulations were measured using a high-precision clinical balance (precision: 0.001 g; n = 6 per formulation; Kensei Kogyo Co., Ltd., Tokyo, Japan) before the experiment. To prevent contamination of clinical equipment, a standard laboratory balance was used for wet weight measurements after immersion.

#### Medium preparation.

Three types of dissolution media were prepared: distilled water (control), xanthan gum-thickened water, and swallowing aid jelly. The jelly (SA-J: Okusuri Nometane, 18 g stick pack; Ryukakusan Co., Ltd., Tokyo, Japan) was an agar-based hydrogel matrix with a water content of 85%–90% and a pH of 6.0–6.5. The thickened water was prepared using three commercially available xanthan gum-based products. TA-O (Tsururinko Quickly; Clinico Co., Ltd., Tokyo, Japan) was the product used in the index case. Two additional products were included for comparison: TA-A (Tromiup Perfect; The Nisshin OilliO Group, Ltd., Tokyo, Japan) and TA-B (Tromi Power Smile; Healthy Food Co., Ltd., Tokyo, Japan). To replicate manual media preparation in clinical settings, the thickeners were manually stirred for dissolution in 100 mL of distilled water. To control for manual variation, 1.0% thickened water solutions were prepared independently by four experimenters. Because the SA-J sample contained insufficient free water to serve as an immersion medium, the sandwich method was adopted as a clinically representative form of administration. The sandwich method, recommended by the Japanese Society of Dysphagia Rehabilitation, involves encasing tablets between two portions of swallowing aid jelly [9]. As MgO tablets tend to float, this procedure ensures complete encapsulation. In this study, the tablet was placed on half of the jelly and covered with the remaining half, and the jelly-encased tablet was then gently immersed in distilled water (S1 Fig).

#### Disintegration test protocol.

A static immersion model was used to simulate esophageal stasis. Unlike standard pharmacopeial tests, which involve mechanical agitation, this method was selected to reproduce a low-shear environment [10]. Briefly, the tablets were immersed in each dissolution medium and maintained at 37 ± 0.5 °C using a constant-temperature water bath (Model BM100; Yamato Scientific Co., Ltd., Tokyo, Japan). A 30-min cutoff was selected to represent a clinically relevant timeframe that exceeded the normal esophageal transit time. For the extended 24-h observations, the beakers were sealed with plastic wrap to prevent evaporation.

#### Measurement protocol.

To ensure measurement objectivity, particularly for the rapid disintegration observed in distilled water, four independent observers recorded disintegration times. The endpoint was defined as the time at which the tablet completely dispersed into fine particles. To minimize interobserver variability, trials were considered unreliable if the difference between the recorded maximum and minimum times exceeded 10%; unreliable trials were repeated. A sample size (n) of 6 per group was selected based on prior disintegration studies in comparable settings [10,11]. As the present study was exploratory, no formal *a priori* power calculations were performed.

#### Operational definitions.

Disintegration outcomes were classified into three categories based on pre-established visual and physical reference standards:

Complete disintegration (CD): The tablet was completely dispersed into fine particles, with no visible solid core remaining by the 30-min cutoff (Fig 1A).

**Fig 1:**
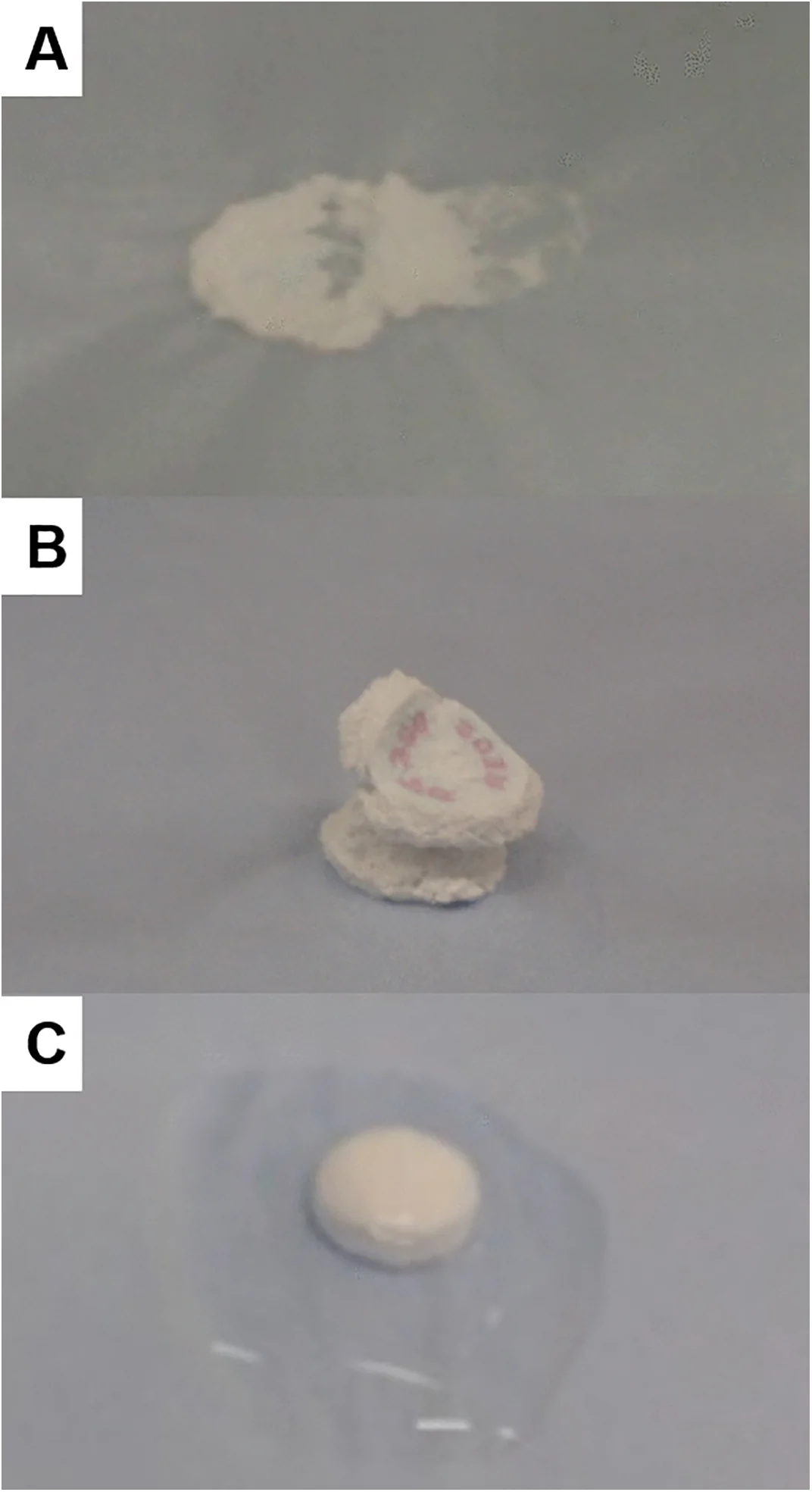
Reference standards for the operational definitions of magnesium oxide (MgO) tablet disintegration behavior. (A) Complete disintegration (CD). The tablet completely dispersed into fine particles with no visible solid core remaining. (B) Friable non-disintegration (FN). The tablet retained its gross morphology but lost its structural integrity and crumbled easily upon minimal mechanical stimulation. (C) Complete non-disintegration (CN). The tablet core remained fully intact, retaining its morphology and mechanical rigidity without visible dispersion.

Friable non-disintegration (FN): The tablet retained its gross morphology by the 30-min cutoff but lost its structural integrity and crumbled easily upon minimal mechanical stimulation (Fig 1B).

Complete non-disintegration (CN): The tablet core remained fully intact and retained its morphology and mechanical rigidity, with no visible dispersion by the 30-min cutoff (Fig 1C).

#### Baseline disintegration test.

The disintegration times in distilled water were measured for all four MgO formulations (F-O, F-1, F-2, and F-3; n = 6 per formulation).

#### Concentration dependence and swelling evaluation.

The disintegration of the index formulation (F-O) was observed by immersing it in the index thickener solution (TA-O) at concentrations of 1.0%, 2.0%, and 3.0% (w/v) (n = 6 per condition). These correspond to stages 1 (50–150 mPa·s), 2 (150–300 mPa·s), and 3 (300–500 mPa·s), respectively, of the Japanese Society of Dysphagia Rehabilitation Dysphagia Diet 2021 classification. To evaluate the long-term physical changes, an additional test was conducted, extending the observation time to 24 h (n = 3 per condition). Tablet weight was measured before and after immersion to quantify the degree of disintegration inhibition and the associated swelling from water absorption.

#### Effect of dispersion media on disintegration.

To compare the influence of different media on tablet disintegration, the index drug (F-O) was tested in the following media: distilled water (control), three thickener solutions at 1.0% (TA-O, TA-A, and TA-B), and SA-J (n = 6 per medium).

#### Inter-formulation comparison.

To confirm whether disintegration inhibition was specific to one MgO formulation or common to all four tested, each was subjected to a static immersion disintegration test in 1.0% TA-O solution (n = 6 per formulation). The *in vitro* disintegration experiments and baseline measurements were conducted at the Department of Pharmacy at Shin-Kuki General Hospital. The facility was maintained under 24-h climate-controlled conditions (temperature range: 23–25 °C; relative humidity: 38%–45%).

### Market price survey

#### Survey design and target regions.

In December 2025, a cross-sectional survey was carried out to assess the retail price and availability of SA-J as an alternative swallowing vehicle in a real-world setting. The survey covered nine geographically distinct locations in Japan: Kuki and Sayama in Saitama; Anjo and Toyokawa in Aichi; Matsumoto in Nagano; Kitakyushu in Fukuoka; Ota and Midori in Gunma; and Isawa in Yamanashi. These sites were selected to represent suburban and regional municipalities, capturing the socio-demographic characteristics of Japan’s aging population. The survey was exploratory and not intended to provide nationally representative epidemiological estimates.

#### Data collection protocol.

To enhance transparency and reproducibility, the survey focused on major drugstore chains and excluded small independent pharmacies. To reflect the sociodemographic landscape of Japan, where older adults frequently care for other older adults, this study focused on physical drugstores rather than online retailers. In these caregiving scenarios, local pharmacies are the most accessible and primary source of medical supplies. This makes retail availability and pricing the most important factors influencing the choice of swallowing aids. A maximum of five representative stores were selected per municipality. Duplicate data from multiple branches of the same chain within a single municipality were removed to establish a single representative price per chain per municipality. Volunteers collected data using a standardized data collection form written in Japanese; an English translation of this form is available in the Supporting Information (S1 File).

#### Data recording and availability definition.

The recorded variables included survey date, store name, product name, package size, and retail price (including tax). Product availability was assessed at the time of the visit. A product was defined as unavailable (stockout) if (1) a shelf tag was present but the product was physically absent or (2) only small-volume packages were available, failing to meet the predefined bulk purchasing criterion (≥800 g for thickeners), which was selected to approximate long-term care usage patterns. Unavailability cases were analyzed descriptively and excluded from the cost comparison analyses.

#### Cost calculation and statistical analysis.

The cost per administration was calculated based on the standard usage amounts: 1.0 g for thickeners and 18 g (equivalent to one sachet) for jellies.

Statistical analyses were performed using SPSS version 24.0 (IBM Corp., Armonk, NY, USA). For the *in vitro* study, values are expressed as the mean ± standard deviation (n = 6 per group). The Kruskal–Wallis test was used to compare disintegration times across multiple formulations, followed by Dunn’s test with Bonferroni correction for post hoc pairwise comparisons. For the market survey, prices are presented as the median and interquartile range (IQR) because of their non-normal distribution, and between-group comparisons were performed using the Mann–Whitney U test. Exact p-values are reported, with statistical significance set at p < 0.05.

## Results

### Clinical case and analysis

#### Radiological and endoscopic findings.

On day 1, a high-attenuation foreign object was detected in the mid-esophagus of the patient during the treatment-planning CT scan (Fig 2A). The object remained at the same location on the setup cone-beam CT performed on day 3 (Fig 2B). Subsequent endoscopy confirmed a brownish residue just above the stricture (Fig 2C), which was carefully removed.

**Fig 2:**
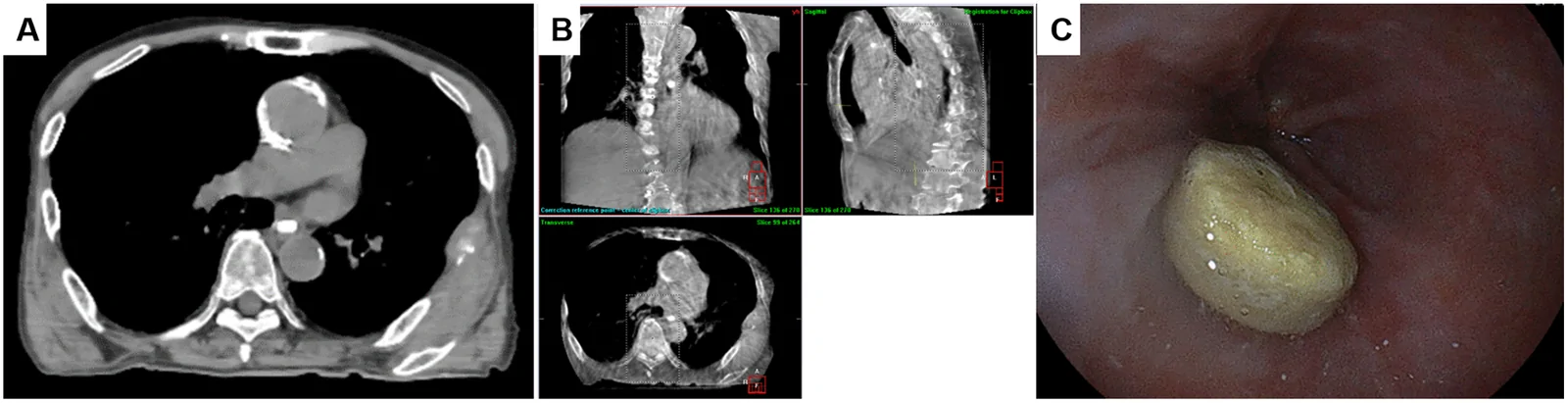
Radiological and endoscopic findings of the esophageal foreign body in the index case. (A) High-attenuation area detected during radiation treatment-planning CT on day 1. (B) The same high-attenuation area on setup cone-beam CT performed on day 3. (C) Endoscopic view of a foreign body in the mid-esophagus, located immediately proximal to the stricture.

#### Identification and physical state of Object X.

After retrieval, the foreign body was found to be encapsulated in a clear, colorless gelatinous film. When this material was removed, the surface of Object X appeared brownish (Fig 3). Object X could not be crushed manually; it was pulverized using a mortar and pestle.

**Fig 3:**
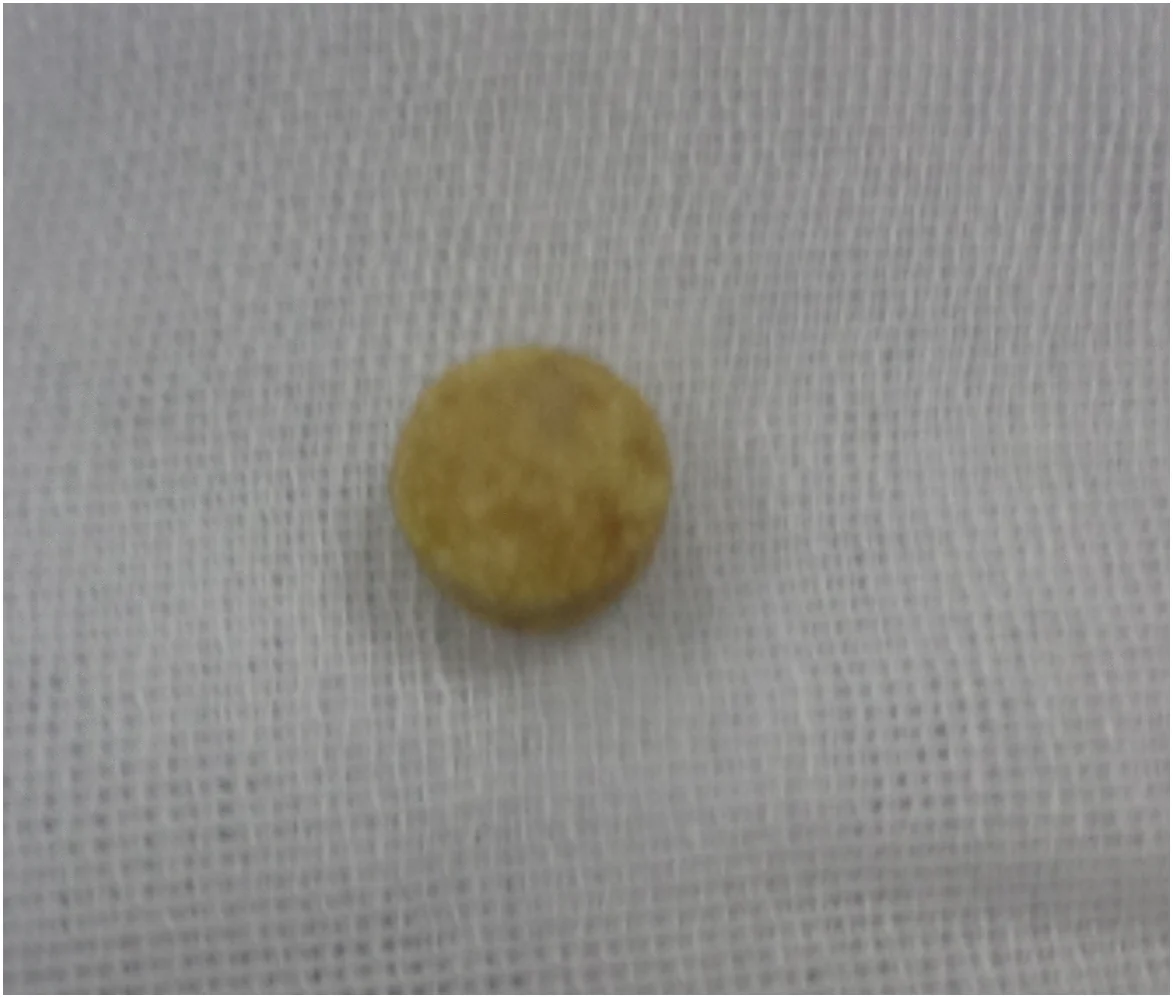
Macroscopic appearance of Object X retrieved from the esophagus. The core surface appeared brownish following manual removal of the encapsulating colorless gelatinous film. Object X was resistant to manual crushing, and pulverization required a mortar and pestle.

#### Dimensions, weight, and radiodensity.

Quantitative measurements revealed that Object X was larger and heavier than the control MgO tablet (S1 Table and S2 Fig). It weighed 500 mg and measured 10 mm in diameter and 5 mm in height (S3 Fig). In contrast, the control dry MgO tablet (330 mg formulation, F-O) weighed 413.7 ± 5.3 mg, with a diameter and height of 8.7 and 4.5 mm, respectively. Other candidate medications were substantially smaller and were excluded on the basis of size.

The mean attenuation value of Object X (856 HU) was lower than that of the dry control MgO tablet (1010 HU) but clearly distinct from those of the other candidate drugs, betamethasone (18 HU) and famotidine (226 HU) (S4 Fig). The observed reduction in CT attenuation (approximately 15%; from 1010 to 856 HU) was consistent with the estimated decrease in density (approximately 18%) derived from the dimensional and weight changes presented in Table 1. These findings support the interpretation that Object X is a swollen, hydrated MgO tablet.

**Table 1:** Comparison of the physical dimensions and weight of Object X, the control magnesium oxide (MgO) tablet, and the other candidate medications.

Sample	Diameter (mm)	Height (mm)	Weight (mg)
Object X	10.0	5.0	500.0
F-O (control)	8.7	4.5	413.7
Famotidine (candidate)	7.3	3.3	150.0
Betamethasone (candidate)	5.0	2.0	46.2

#### Chemical analysis and final identification.

Neither the intact nor the halved Object X exhibited visible disintegration after immersion in distilled water (Fig 4). The control MgO tablet dissolved almost completely under identical conditions. Object X produced a substantial amount of white precipitate only after crushing (Fig 5). The Mg concentration in the supernatant was markedly lower in the Object X samples (0.51 ± 0.03 mg/dL; 2.04 mg/dL per g of sample) than in the control solution (1.52 ± 0.04 mg/dL; 7.34 mg/dL per g of sample). In addition, the levodopa colorimetric reaction was positive, indicating alkalinity. Together with the findings above, this is consistent with Object X being a swollen MgO tablet (Fig 6).

**Fig 4:**
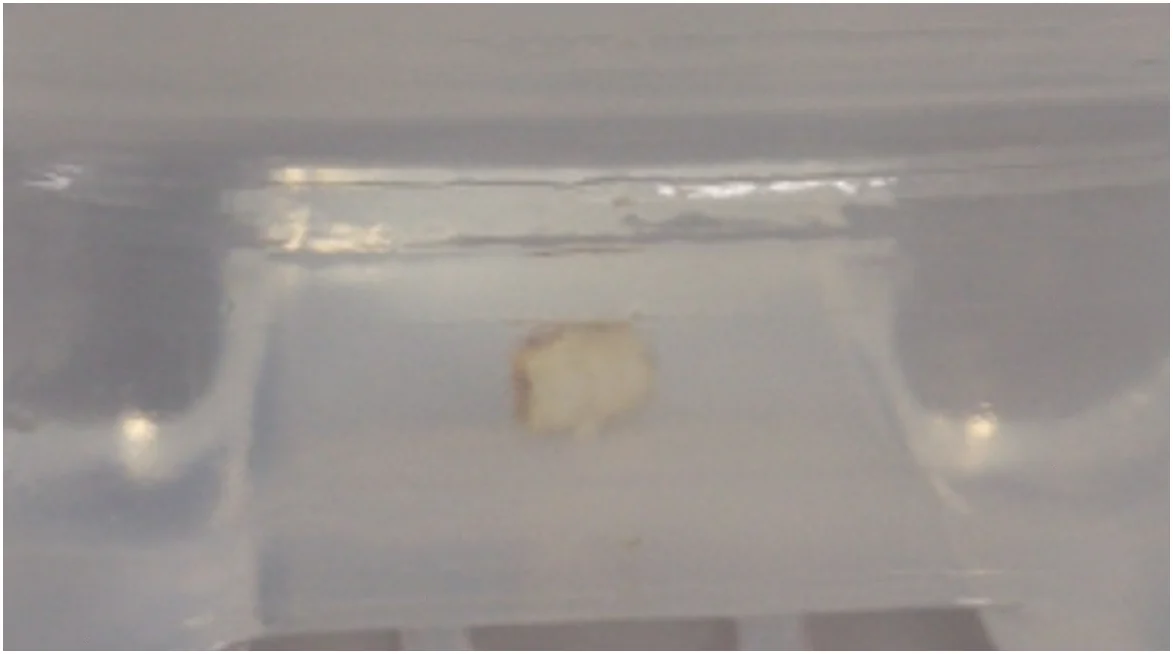
Immersion behavior of intact and halved Object X in distilled water. Both the intact and halved Object X remained physically rigid without any signs of disintegration or dissolution.

**Fig 5:**
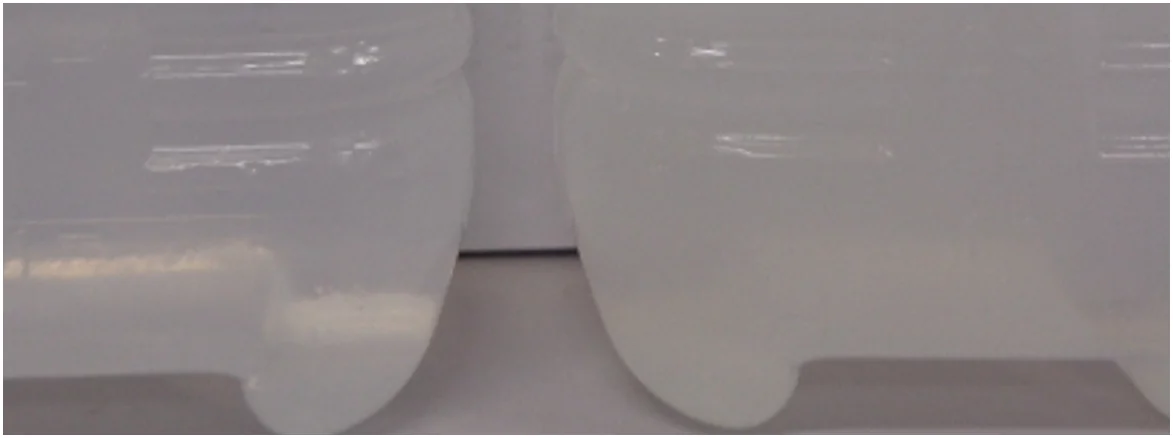
Comparison of dispersion behavior after mechanical pulverization. After mechanical pulverization using a mortar and pestle, Object X and a magnesium oxide (MgO) tablet were subjected to vigorous stirring in distilled water. Object X (left) produced a substantial amount of white precipitate, whereas the control MgO tablet (F-O, right) nearly completely dispersed under identical conditions.

**Fig 6:**
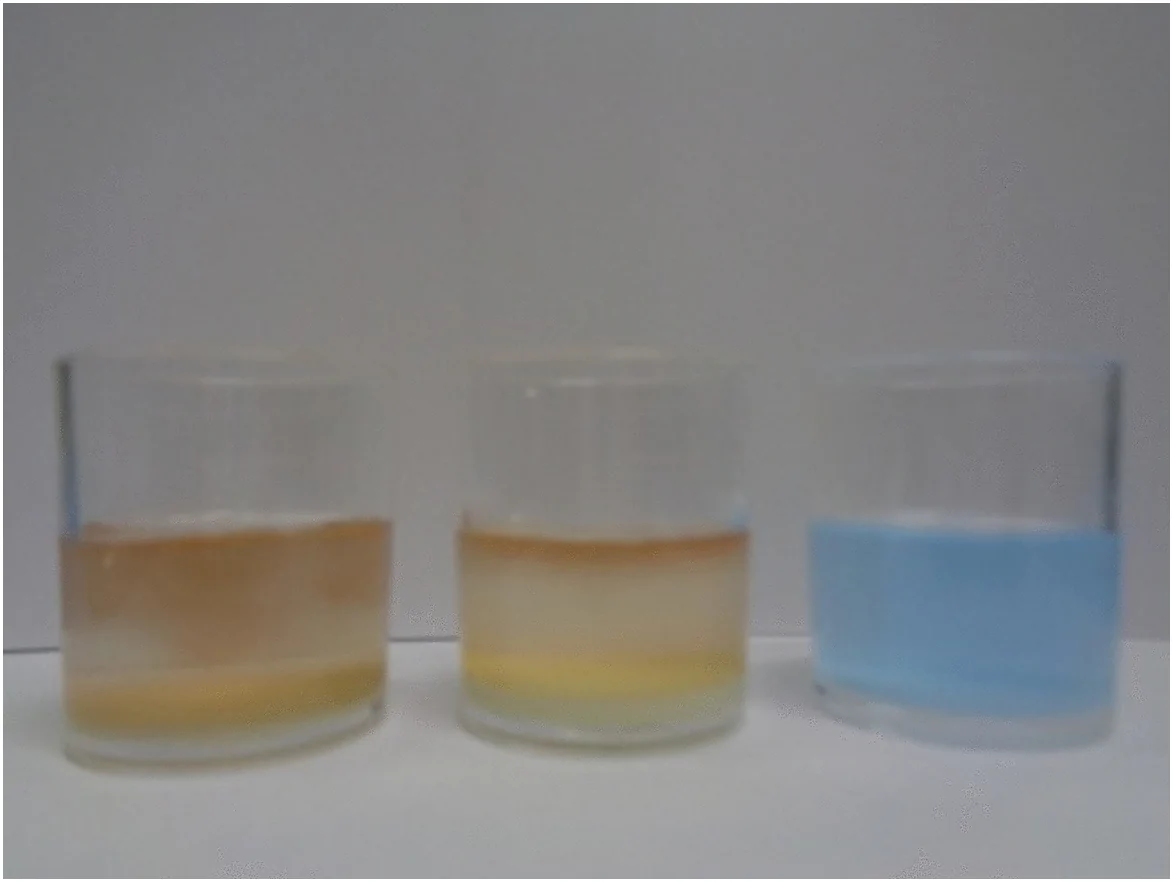
Qualitative alkalinity test using the levodopa colorimetric reaction. One-quarter of each of three tablets—Object X (125 mg), a control MgO tablet (F-O; 100 mg, equivalent to one-quarter tablet), and a levodopa tablet (50 mg), from left to right—was pulverized and suspended in 50 mL of distilled water. Each suspension was then combined with a levodopa solution (50 mg in 50 mL of distilled water) to obtain a final volume of 100 mL. The images show the samples after 1 h of incubation, revealing the characteristic browning indicative of an alkaline reaction in Object X and the MgO tablet.

### *In vitro* mechanistic study

#### Baseline physicochemical properties.

The disintegration times of all four MgO formulations were measured in distilled water (0% thickener) (Table 2).

**Table 2:** Baseline disintegration times of the four magnesium oxide (MgO) formulations in distilled water.

Formulation	Disintegration time (s)
F-O (Index)	7.3 ± 0.4
F-1	8.5 ± 1.0
F-2	22.4 ± 0.8
F-3	8.3 ± 0.7

Disintegration values are expressed as mean ± standard deviation.

Although all formulations disintegrated, significant differences were observed in disintegration times (H = 16.759, p < 0.001). Post hoc analysis revealed that F-2 exhibited a significantly longer disintegration time (22.4 ± 0.8 s) than the index formulation F-O (7.3 ± 0.4 s; p < 0.001); no significant differences were detected in any other pairwise comparisons.

#### Concentration-dependent disintegration failure and swelling.

To evaluate the effect of the thickener concentration on disintegration, F-O was immersed in TA-O solutions at 1.0%, 2.0%, and 3.0% (n = 6 per condition). The tablets did not disintegrate within the 30-min observation period at any concentration. However, extended observations over 24 h (n = 3 per condition) revealed that tablets in the 1.0% solution exhibited an FN state, retaining their gross morphology but easily crumbling when handled manually, precluding reliable weight measurements. In contrast, the tablets in the 2.0% and 3.0% TA-O solutions remained in the CN state after 24 h, preserving their shape and hardness. The weight of the tablets increased by approximately 2.2-fold, from 0.414 ± 0.005 g to 0.91 ± 0.02 g and 0.92 ± 0.01 g for the 2.0% and 3.0% TA-O solutions, respectively.

#### Disintegration by media type.

F-O was tested in three thickener solutions (TA-O, TA-A, and TA-B) at 1% concentration and in SA-J (n = 6 per medium). F-O did not disintegrate (CN state) in any of the thickener solutions by the 30-min cutoff. In contrast, the tablets embedded in SA-J disintegrated (CD state) within 25.4 ± 1.7 s.

#### Inter-formulation comparison.

The disintegration of all four MgO formulations was tested in a 1.0% TA-O solution (n = 6). None of the formulations disintegrated (CN state) at the 30-min cutoff.

### Market price and availability survey

Market price and availability survey data were obtained for 43 thickener products and 43 jelly products (S1 Data and S2 Data). After excluding two duplicates from each group, the final analysis set included 41 thickeners and 41 jellies. Unavailability was observed for five thickeners (12.2%) and six jellies (14.6%). Based on the remaining products available for cost calculation (36 thickeners and 35 jellies), the median cost per single administration was 4.3 JPY (0.03 USD; IQR: 4.2–4.3 JPY) for thickeners and 62.5 JPY (0.41 USD; IQR: 62.5–62.9 JPY) for jellies. This represents an approximately 14.5-fold higher cost for SA-J than for thickeners (p < 0.001). Conversions use an exchange rate of 152.4 JPY/USD as of December 2025.

## Discussion

This study addressed a critical but often overlooked safety issue in dysphagia management: the potential hazard of thickening agents causing iatrogenic mechanical obstruction due to unintended interactions with oral medications. Our investigation was prompted by a clinical case in which a standard MgO tablet failed to disintegrate and formed a rigid mass within a malignant esophageal stricture. Through a combination of retrospective case analysis, *in vitro* experiments, and a market survey, we successfully characterized this phenomenon and identified the structural barriers to its prevention.

The present study revealed three primary findings: (1) Object X extracted from the esophagus of the index case was identified as a non-disintegrated F-O tablet encapsulated by TA-O; (2) xanthan gum-based thickeners consistently inhibited the disintegration of MgO tablets across all tested formulations and thickener brands, resulting in a rigid mass formation; and (3) SA-J did not impede the rapid disintegration of F-O *in vitro*. In this study, we used the term “hazard” in its epidemiological sense (a potential source of harm or adverse condition) to refer to the intrinsic physicochemical tendency of MgO tablets to form rigid masses with xanthan gum-based thickeners. We avoided the use of the term “risk,” as conducting a formal risk assessment would necessitate quantitative probability estimates, which are beyond the scope of the present data. The three primary findings are discussed in detail below.

### Finding 1: Identification of the extracted Object X

Object X extracted from the malignant esophageal stricture in the index case was definitively identified as a non-disintegrated F-O tablet encapsulated by a residual TA-O gel layer. The ex vivo CT attenuation (856 HU) and physical dimensions (10.0 mm diameter, 500.0 mg weight) were consistent with a hydrated, swollen MgO core. Furthermore, analyses after pulverization—specifically the reduced supernatant Mg concentration and positive alkaline levodopa colorimetric reaction—confirmed its chemical identity as MgO. While a 10-mm mass typically traverses an unobstructed digestive tract, in the presence of severe malignant stricture such an insoluble and hardened mass poses a critical physical hazard of mechanical esophageal obstruction [12,13].

### Finding 2: Disintegration inhibition and rigid mass formation by thickeners

Complete non-disintegration (CN state) was observed for all four tested MgO formulations in the index thickener (TA-O), and the index formulation (F-O) consistently exhibited the CN state across all three tested xanthan gum-based thickeners (TA-O, TA-A, and TA-B). Furthermore, at 2.0–3.0% concentrations, tablet weight increased by approximately 2.2-fold, resulting in rigid masses resistant to manual crushing.

This disintegration arrest and hardening can be explained by a two-step physicochemical mechanism: (1) water sequestration by the xanthan polymer network limits free-water availability, impairing capillary penetration into the tablet core [11,14]; and (2) divalent magnesium ions (*Mg*^2+^) released at the interface bind to carboxyl groups on xanthan gum side chains, forming ionic crosslinks (junction zones) that create an insoluble gel layer on the tablet surface [15]. This barrier blocks further water ingress, leaving the tablet swollen and hardened.

### Finding 3: Preservation of rapid disintegration by SA-J and its clinical/economic implications

In contrast, SA-J did not impede the rapid disintegration of F-O *in vitro*; complete disintegration occurred within 25.4 ± 1.7 s, comparable to that in distilled water. Because SA-J is an agar-based hydrogel matrix rather than a xanthan gum-based product, it does not form an ionic crosslinking barrier and readily releases water across the tablet interface. In high-volume clinical and nursing care settings, delays between medication preparation and administration are common. This property may therefore be clinically important, because the retained flexibility of the jelly would allow bolus deformation and peristaltic clearance through the esophagus. However, the market survey showed that the per-dose cost of SA-J is approximately 14.5-fold higher than that of bulk thickeners, which presents a substantial economic barrier to its routine adoption. The structural reasons why this hazard remains latent in clinical systems are discussed below.

#### Clinical blind spot.

We further explored why the significant hazard of MgO disintegration arrest remains unnoticed despite its clinical relevance. First, we considered that this phenomenon is clinically invisible in most cases. The solid mass (approximately 10 mm) retrieved in our study would likely pass through the gastrointestinal tract asymptomatically, unless there is passage impairment, such as severe stricture or achalasia. Even when the laxative effect is diminished or absent, potentially owing to the physicochemical sequestration of MgO within the xanthan gum matrix, this clinical non-response is often interpreted as drug resistance. This misinterpretation can lead to dose escalation or switching to alternative medications. However, the medication vehicle itself is seldom suspected, a classic form of cognitive error [16]. This systemic failure arises from a convergence of clinical and structural barriers: information silos between prescribers and caregivers obscure the co-administration practice, institutional financial inertia favors inexpensive thickeners over safer alternatives, and regulatory gaps between pharmaceuticals and food products preclude mandatory cross-compatibility testing. Consequently, these intertwined barriers allow a critical physicochemical hazard—the arrested disintegration and rigid mass formation of MgO—to remain entirely latent and unaddressed in routine care. Notably, a second case encountered during the preparation of this manuscript (S5 Fig) suggests that the identified hazard may not be unique to the index case.

#### Barrier 1: Information silos.

A structural division exists between prescription, dispensation, and administration processes, creating conditions in which critical safety information fails to reach those responsible for medication administration. Physicians, pharmacists, and caregivers each operate within distinct professional frameworks, and the co-administration of thickeners with oral medications often occurs outside the awareness of the prescriber or dispensing pharmacist [4]. This fragmentation obscures the unintended physicochemical hazards arising from the combination of MgO and thickeners. It may also delay recognition of adverse events—particularly in patients with cognitive impairment who cannot communicate discomfort.

#### Barrier 2: Economic disincentives and institutional inertia.

The most significant barrier to the widespread use of SA-J, a potentially safer alternative, is economic. Our market survey revealed that the median cost per administration of SA-J was approximately 14.5-fold higher than that of commercially available xanthan gum-based thickeners. Within the Japanese healthcare insurance framework, the expenses for swallowing-aid vehicles are not separately reimbursable and are often bundled into institutional operating costs. This substantial economic disparity acts as a powerful financial deterrent against adopting SA-J, effectively compelling facilities to rely on inexpensive, bulk-packaged xanthan gum-based thickeners. Furthermore, cost-oriented practices established in inpatient settings are often uncritically transferred to home medical care, further reinforcing the use of thickeners as the default option.

#### Barrier 3: Regulatory gap.

A structural regulatory gap further exacerbates the latent hazard. As a pharmaceutical, MgO is regulated by the Pharmaceuticals and Medical Devices Act in Japan [17], whereas thickeners are classified as food under the Food Sanitation Act [18]. Consequently, testing the interaction between these products is not mandatory. The practical consequences became evident during the clinical treatment of the index case: when the treating physician contacted the thickener manufacturer for guidance, the response was that interaction testing was not legally mandated for food products. A follow-up with the MgO manufacturer revealed that conducting cross-tests against all food products was not economically feasible. This highlights a regulatory blind spot where verifying the safety of interactions between pharmaceuticals and food-classified products used for medical applications is not required prior to market distribution.

### Future perspectives and systemic recommendations

#### Trade-off between safety and economic rationality.

Ensuring the safety of physically vulnerable older patients involves a trade-off with economic reality. Although a complete transition to SA-J would be ideal from a safety standpoint, a more practical approach is to screen high-risk patients through multidisciplinary collaboration and selectively switch their swallowing vehicles to safer options. These include swallowing aid jellies, liquid magnesium hydroxide formulations, and alternative osmotic laxatives that do not interact with xanthan gum.

#### Discretionary power of clinicians and uncertainty of additives in generics.

Clinicians often lack full discretionary control over the specific formulations of dispensed medication. Under the Japanese health insurance system, physicians can select “brand-name or generic” on electronic medical records; however, they usually cannot specify the manufacturer of the generic dispensed at pharmacies [19]. Although cost containment drives this policy, it complicates the management of additive-related differences in physicochemical drug behavior [20]. Even when the active ingredients are identical, the excipients, binders, disintegrants, and coatings vary by manufacturer [21]. These differences affect the disintegration and dissolution of drugs in water and can interact unpredictably with food matrices or thickeners [22]. In our study, the inter-formulation comparison revealed subtle but distinct differences: formulation F-2 generated coarser aggregate particles and formed a thin powder film on the water surface during disintegration (S6 Fig), in contrast to formulations F-1 and F-3. These differences may influence clinical outcomes, including the site of impaction in strictures and the difficulty in endoscopic removal. Therefore, careful consideration of these additive effects is warranted in future medication management guidelines.

#### Lack of unified safety standards for drug–food interaction disclosure.

Finally, no unified infrastructure exists for disclosing the physicochemical interactions between pharmaceuticals and food-classified swallowing aids. One potential approach would be the development of a standardized disclosure mechanism, tentatively termed “Biological SDS”—analogous to industrial Safety Data Sheets—to support team-based medication safety management in clinical and long-term care settings [23].

#### Clinical significance and methodological justification.

The physicochemical incompatibility observed between MgO and xanthan gum-based thickeners aligns with established ion-crosslinking mechanisms [15], confirming that the observed phenomenon is of an intrinsic chemical nature rather than a physical artifact of tablet morphology. Previous studies reported that thickening agents delay the dissolution of drugs such as amlodipine, atenolol, carbamazepine, and warfarin [5]. However, the CN state of MgO observed in this study is a phenomenon fundamentally distinct from the mere “delay” described in the previous report. Conventional disintegration tests based on pharmacopoeia standards are conducted under conditions that involve vigorous mechanical agitation and high shear forces [10]. Therefore, these methods may underestimate tablet behavior in a static stagnation state within the esophagus. Notably, the “delayed disintegration” in high-viscosity media reported in previous studies may include experimental artifacts derived from aggressive test conditions. This should be distinguished from the CN state observed in our study. In this study, we prioritized physiological validity over compliance with standard regulatory conditions and adopted a static immersion model. Under this model, the disintegration process was not merely delayed but completely arrested. Moreover, this static model is useful for physicochemical hazard characterization in the most vulnerable patients, especially those with predominant stasis, such as patients with severe strictures or achalasia.

#### Limitations.

This study has certain limitations that must be acknowledged. First, the clinical aspect of this study was based on a single index case presenting with esophageal stricture. The complete mechanical obstruction seen in this patient is unlikely to occur in individuals with normal gastrointestinal anatomy. Moreover, the epidemiological frequency of this adverse event in the general elderly population could not be estimated solely from this observational data. Therefore, our findings should be interpreted as highlighting a physicochemical hazard, an intrinsic characteristic of the drug–thickener combination, rather than quantifying the clinical risk, which would require prospective epidemiological studies and comprehensive risk assessment modeling. Second, our *in vitro* model used a static immersion method instead of a standard pharmaceutical dissolution tester. This choice was intentional, as the low-shear static model was designed to simulate the physiological state of esophageal stasis, which standard agitation-based methods cannot replicate. However, this model did not entirely replicate the completely hardened mass (Object X) observed in the index case, suggesting that additional *in vivo* factors, such as prolonged contact time, body temperature, or peristaltic compression, may further potentiate gel dehydration and core hardening. Therefore, the static model should be considered a conservative estimate of the hazard under static conditions rather than a complete mechanistic simulation of the clinical event. This is because the model does not replicate dynamic esophageal factors, such as gravitational effects, mucosal secretions, and intermittent peristaltic contractions. Furthermore, the comparative evaluation between SA-J and xanthan gum-thickened water involved inherent differences in physicochemical parameters, including water content, pH, rheological properties, and media volume (18 g jelly encapsulation vs. 100 mL solution). This experimental design was intentionally chosen to replicate standardized real-world clinical administration protocols (e.g., the sandwich method recommended by clinical guidelines) rather than using purely equivalent fluid environments. Future studies with strictly controlled physicochemical variables would provide further mechanistic clarity. Third, although the thickener solutions were prepared by four independent experimenters to assess robustness against manual variation, their viscosity was not measured instrumentally because the appropriate rheological equipment was not available in our clinical setting. Similarly, instrumental rheological measurements of SA-J were not performed, as appropriate equipment was unavailable and no pharmaceutical package insert exists for this food-classified product. However, the complete concordance of outcomes across all preparers and replicates (100% non-disintegration in all thickener conditions) suggests that the observed effect was resilient to minor concentration variances. The preparation conditions nonetheless reflect real-world conditions in clinical and home care settings, where thickeners are mixed by hand without any instrumental verification. Fourth, a sample size of n = 6 per group was determined empirically based on prior disintegration studies, and no formal *a priori* power calculations were performed. Although the non-disintegration (CN state) observed across all experimental conditions supports the adequacy of this sample size, this was not established prospectively. Future studies using formal sample size justifications would strengthen the statistical basis of our findings. Fifth, we focused on macroscopic disintegration behavior and did not use microscopic imaging techniques, such as scanning electron microscopy, to characterize the polymer–drug interface. Although our findings are consistent with the established physicochemical principles regarding xanthan gum and water sequestration, the proposed mechanism remains a macroscopic inference rather than a molecularly verified model. Sixth, the market price survey was limited to cross-sectional sampling across nine regions and was exploratory in nature. A comprehensive pharmacoeconomic analysis that incorporates insurance reimbursement structures would be needed to establish nationwide cost patterns. Finally, regarding the *ex vivo* CT analysis, the attenuation value of the betamethasone tablet (18 HU) was likely underestimated owing to the partial volume effect, as the tablet dimensions were below the spatial resolution of the CT scanner. However, given the decisive dimensional discrepancy between betamethasone and Object X, this limitation did not affect the identification process.

## Conclusion

Although based primarily on a single index case and therefore requiring cautious interpretation, our findings indicate that the combination of MgO tablets and xanthan gum-based thickeners can lead to an underrecognized arrest of tablet disintegration. This phenomenon was consistently reproduced in each formulation and thickener combination tested. While SA-J avoided this effect in vitro, its clinical implementation is limited by structural barriers, including substantial economic considerations. In an increasingly complex medication-use environment, recognizing that “thickened water is not equivalent to water” represents an essential first step toward ensuring patient safety. Future research should focus on quantifying the epidemiological risk within the general elderly population and developing dynamic physiological models to better simulate *in vivo* conditions.

## Supporting information

S1 Fig Sandwich method for experimental preparation. Photograph of the MgO tablet encased in swallowing aid jelly (SA-J).
(TIF)

S2 Fig Macroscopic comparison of candidate medications and Object X. Representative photographs showing the visual comparison between the index mass and candidate tablets.
(TIF)

S3 Fig Quantitative measurement of the dimensions of Object X. Dimension data and measurement methodology for the retrieved mass.
(TIF)

S4 Fig Comparative *ex vivo* CT imaging of candidate medications and Object X. CT images comparing internal structure and radiodensity among formulations.
(TIF)

S5 Fig Recurrence of the same phenomenon in an independent clinical case. Recurrence of a similar phenomenon observed in a second clinical case.
(TIF)

S6 Fig Coarse particles and the surface film observed in F-2. Macroscopic appearance and surface characteristics of the non-disintegrated residue.
(TIF)

S1 Table Physical consistency between the reduction in density and CT attenuation. Summary table comparing calculated density with measured CT attenuation values.
(DOCX)

S1 Data Market survey data for thickener products. Tabulated market data of commercial xanthan gum-based thickeners.
(CSV)

S2 Data Market survey data for swallowing aid jelly products. Tabulated market data of the commercial swallowing aid formulations.
(CSV)

S1 File Market price survey data sheet regarding medication assistance vehicles. (DOCX)
