# Supplementary material for: Magnesium oxide tablets form rigid masses in xanthan gum-based thickeners: A physicochemical hazard revealed by a clinical case
Source: PLoS One. 2026 Oct 29;21(10):e0361494. doi: 10.1371/journal.pone.0361494 (PMC13718963; doi:10.1371/journal.pone.0361494)
Supplement: Supplementary file 1 [file pone.0361494.s007.docx]

**S1 Table**. Physical consistency between density decrease and CT attenuation reduction

| **Parameter** | **MgO Control (Dry)** | **Object X (Recovered)** | **Change (%)** |
| --- | --- | --- | --- |
| **Weight (mg)** | 413.7 | 500.0 | +20.9% |
| **Diameter (mm)** | 8.7 | 10.0 | +14.9% |
| **Height (mm)** | 4.5 | 5.0 | +11.1% |
| **Calculated Volume (mm³)** | 267.5 | 392.7 | +46.8% |
| **Calculated Density (mg/mm³)** | 1.55 | 1.27 | -18.1% |
| **Mean CT Attenuation (HU)** | 1010 | 856 | -15.2% |

MgO, magnesium oxide; CT, computed tomography.
