# Supplementary material for: Magnesium oxide tablets form rigid masses in xanthan gum-based thickeners: A physicochemical hazard revealed by a clinical case
Source: PLoS One. 2026 Oct 29;21(10):e0361494. doi: 10.1371/journal.pone.0361494 (PMC13718963; doi:10.1371/journal.pone.0361494)
Supplement: Supplementary file 2 [file pone.0361494.s010.docx]

**S1 File. Data Collection Instrument**

**English Translation**

**Market Price Survey Data Sheet regarding Medication Assistance Vehicles**

**Surveyor Name: ____________________ Assigned Area (City): ____________________**

**■ Purpose of Survey: This is an academic survey to clarify the economic burden of medication management in older adults. Thank you for your cooperation.**

**【Important Rules for Survey】**

**The data collected in this survey will serves as baseline information for academic research. Please observe the following rules strictly.**

- **Target Stores: Please target only major drugstore chains (e.g., Matsumoto Kiyoshi, Welcia, Sugi Pharmacy).**
  o ***Note:* Please exclude small privately owned pharmacies.**
  o ***Note:* If there are multiple stores of the same chain within the assigned city (e.g., three "Pharmacy A" stores in Kuki City), please survey only one store (to prevent duplicate counting).**
- **Target Products:**
  o **【A】 Thickener (Powder)**
     **Target: Large capacity pack (containing approx. 800 g–1200 g)**
     **Priority Product: *Tsururinko Quickly* (Clinico)**
     **If "Tsururinko" is unavailable: Please record one of the products from the list below:**
       ***Toromi-Up Perfect* (Nisshin Oillio)**
       ***Meiji Kantan* Toromake (Meiji)**
       ***Yasashii Kondate Toromi Fine* (Kewpie)**
       ***Toromina Soft Type***
       ***Torominar***
     **If none of these are available: Check the "Ingredients" on the back of the package; any product listing "Polysaccharide thickener (Zonen-tatorui)" is acceptable regardless of the manufacturer (*Note: Please avoid older types listing "Starch"*).**
  o **【B】 Swallowing Aid Jelly**
     **Target: Single-use stick type (approx. 6 sticks per box)**
     **Products to look for: *Okusuri Nometane* (Ryukakusan), etc.**
     ***Note:* Flavor (grape, strawberry, etc.) is not important.**
- **Recording Prices: Please ensure that you record the "Tax-included Price.”**
  o ***Note:* Please exclude items with "Clearance" or "Markdown" stickers (as this is a survey based on the list prices).**
  o **Do not apply coupons, membership discounts, or point rebates (the list price on the shelf is acceptable).**
- **Regarding Photography: As many stores prohibit indoor photography, do not insist on taking photos; please perform handwritten recording onto this sheet.**

**Survey Sheet A: Thickener (Target: "800 g–1200 g" Pack)**

| **Survey Date** | **Chain Store Name** | **Product Name** | **Content** | **Selling Price (Tax inc.)** |
| --- | --- | --- | --- | --- |
|  |  |  | G | ¥ |
|  |  |  | G | ¥ |
|  |  |  | G | ¥ |
|  |  |  | G | ¥ |
|  |  |  | G | ¥ |

**Survey Sheet B: Swallowing Aid Jelly (Okusuri Nometane)**

| **Survey Date** | **Chain Store Name** | **Product Name** | **Content** | **Selling Price (Tax inc.)** |
| --- | --- | --- | --- | --- |
|  |  |  | g | ¥ |
|  |  |  | g | ¥ |
|  |  |  | g | ¥ |
|  |  |  | g | ¥ |
|  |  |  | g | ¥ |

**【Entry Example】**

| **Survey Date** | **Chain Store Name** | **Product Name** | **Content** | **Selling Price (Tax inc.)** |
| --- | --- | --- | --- | --- |
| Dec 11 | Chain S Drugstore Kuki | Toromi-Up | 800 g | ¥1200 |
| Dec 11 | Chain O Pharmacy Kuki | Tsururinko | 1000 g | ¥3900 |
| Dec 13 | Chain I Drug Kuki | Out of Stock | - | - |
| Dec 14 | Chain C Healthcare Kuki | Toromina Soft Type | 800 g | ¥1400 |
| Dec 15 | Chain H Chemist Kuki | Toromi Fine | 1200 g | ¥2980 |

Remarks: At Chain C Healthcare Kuki, only the strawberry flavor cost 990 yen. At Chain I Drug Kuki, the product was out of stock. The 12-pack version costs 2000 yen.
