# Supplementary material for: Magnesium oxide tablets form rigid masses in xanthan gum-based thickeners: A physicochemical hazard revealed by a clinical case
Source: PLoS One. 2026 Oct 29;21(10):e0361494. doi: 10.1371/journal.pone.0361494 (PMC13718963; doi:10.1371/journal.pone.0361494)
Supplement: Supplementary file 3 [file pone.0361494.s012.docx]

**Response to Academic Editor and Journal Requirements**

Dear Dr. Claudio J. Salomon,

Academic Editor, PLOS ONE

Manuscript ID: PONE-D-26-27019

Title: Magnesium oxide tablets form rigid masses in xanthan gum-based thickeners: A physicochemical hazard revealed by a clinical case

We sincerely thank the editorial team for handling our manuscript. We have addressed all Journal Requirements and editorial requests as detailed below.

Response to Journal Requirement

1. Style & Formatting Requirements

Journal Requirement: Ensure manuscript meets PLOS ONE style and file naming requirements.

Response:

We have thoroughly reviewed the manuscript and all accompanying files against PLOS ONE formatting guidelines and official style templates:

In-text citations for figures, tables, and supporting files have been standardized to PLOS ONE formatting.

All figure files have been renamed strictly following official specifications (Fig1–Fig6, S1_Fig–S6_Fig).

2. Image Copyright & Licensing

Journal Requirement: Resolve copyright permissions under the CC BY 4.0 license for Figures 1–6, S1–S6, and File S1.

Response:

We have confirmed and resolved the copyright status across all figures and supporting files:

- S1 File (Data Collection Instrument) has been revised to remove external product reference images, ensuring that the entire file consists exclusively of original forms and textual descriptions prepared by the authors.

We explicitly confirm that all figures, photographs, CT reconstructions, schematics, and supporting files (Figs1–6, S1–S6_Figs, S1 Table, S1 File, and S2 File) are original works generated, photographed, and prepared solely by the authors for this study. None of these materials are reproduced or adapted from external publications, websites, or commercial brochures.

As the sole copyright holders of all remaining materials, the authors grant full permission for all figures, tables, and supporting files to be published under the Creative Commons Attribution 4.0 International (CC BY 4.0) License.

3. Supplementary Figure File Types & Legends

Journal Requirement: Amend file type for supplementary figures from 'Figure' to 'Supporting Information' and list legends after the references list.

Response:

In the Editorial Manager submission system, the file type for all supplementary figures (S1_Fig through S6_Fig) and supporting files has been updated to "Supporting Information".

Complete, detailed captions and legends for all Supporting Information items (S1_Fig–S6_Fig, S1 Table, S1 File, and S2 File) are provided in a dedicated section immediately following the References list in the revised manuscript.

4. Recommended Citations

Journal Requirement: Review any recommended citations in reviewer comments and evaluate their relevance.

Response: We have carefully reviewed all literature references suggested in the reviewer comments. No specific citation recommendations were made by either reviewer in this submission. All references cited in the revised manuscript have been independently evaluated for relevance and accuracy.

Response to Reviewer #1

Dear Reviewer #1,

We sincerely appreciate your review and comments on our manuscript (PONE-D-26-27019). We have carefully addressed each of your points and revised the manuscript accordingly. Point-by-point responses are provided below.

1. Clarity and Detailed Explanation

Reviewer Comment:

*"reviewed need clarity explination"*

Response:

We have substantially revised the Abstract, Introduction, and Discussion to more clearly explain the physicochemical mechanism of disintegration inhibition, the clinical relevance of esophageal impaction, and the overall study framework.

2. Availability of Figures, CT Images, and Clinical Data

Reviewer Comment:

*"no images found/ no clinical data evidence and CT"*

Response:

We apologize for the confusion regarding the availability of figures and CT images. In the initial submission, figure files were inadvertently uploaded within .docx wrappers rather than as standalone high-resolution image files, which caused rendering and display issues within the review system.

To fully resolve this issue:

- Primary Figures and CT Images: All primary figures (Figs 1–6) have been converted to high-resolution TIFF files strictly following PLOS ONE specifications (Fig1.tif–Fig6.tif). These include the in vivo clinical CT findings of the impacted mass (Fig 2A and 2B) and the endoscopic confirmation (Fig 2C).
- Supplementary Figures: All supplementary files — including the comparative ex vivo CT imaging (S4_Fig) and the recurrent case CT and clinical data (S5_Fig) — have been updated to standalone TIFF formats and reclassified under the "Supporting Information" category (S1_Fig.tif–S6_Fig.tif). Detailed captions for all Supporting Information items have been placed directly following the References section in the revised manuscript.
- Clinical Evidence: Clinical data and CT evidence from the index case and the recurrent case serve as the clinical foundation of this study, complemented by systematic in vitro replication experiments.

3. Administration Protocol and In Vitro Methods

Reviewer Comment:

*"clearity method of adminstration protocol"*

Response:

We would like to clarify that no prospective drug administration to human subjects was performed for the experimental series. To ensure full transparency and reproducibility, the detailed protocol for the *in vitro* static immersion model (simulating esophageal stasis) and the clinical administration sandwich method used for swallowing aid jelly are documented in full detail in the Materials and Methods section.

4. English Grammar and Linguistic Quality

Reviewer Comment:

*"several grammatic and lingustic errors"*

Response:

The manuscript has undergone comprehensive professional English language editing by Editage ([www.editage.com](https://www.google.com/search?q=https%3A%2F%2Fwww.editage.com)) prior to resubmission. A formal certificate of English editing is provided as a supplementary document. In accordance with PLOS ONE guidelines, we have declared the use of generative AI assistance (Gemini, Google LLC) for initial draft translation and syntactic structuring in the dedicated declaration section, after which all content was verified and refined by the authors and professional editors.

5. Plagiarism and Originality Check

Reviewer Comment:

*"Plagarism check require"*

Response:

We confirm that this manuscript represents original research and has been checked to ensure academic integrity. All figures, photographs, CT scans, schematics, and text are original works created by the authors specifically for this study. The third-party material previously included in File S1 has been completely removed to ensure full compliance with CC BY 4.0 licensing.

Response to Reviewer #2

Dear Reviewer #2,

We sincerely thank the reviewer for the thorough, constructive, and insightful evaluation of our manuscript (PONE-D-26-27019). The comments have helped us significantly improve the scientific precision, methodological transparency, and clinical contextualization of our work.

We have carefully addressed all points raised by the reviewer. Below are our point-by-point responses to the comments and details of the corresponding revisions made to the manuscript.

General Comments:

*"The study contains a potentially important clinical and pharmaceutical observation and could be suitable for publication after substantial revision. The principal issue is not the experimental observation itself, which appears interesting and reproducible, but the extent to which the authors generalize the findings from a single clinical case and a static in vitro model to a broader population-level clinical hazard. Strengthening the methodological description, moderating the causal language, clarifying the mechanism, and providing a more rigorous economic analysis would considerably improve the manuscript."*

Response:

We are grateful for these encouraging and constructive overarching remarks. We agree that our initial framing over-generalized the clinical findings beyond the immediate empirical scope. In the revised manuscript, we have moderated our causal language and avoided overgeneralization throughout, clarified that our study characterizes an intrinsic physicochemical *hazard* under static stasis conditions rather than a quantified population *risk*, expanded the methodological and mechanistic details, and presented the retail market economic survey with appropriate caveats.

Comment 1:

*"1. The statement that xanthan gum-based thickened water “constitutes a hazard embedded in current clinical systems” is stronger than the evidence presented. The study demonstrates that xanthan gum thickeners can markedly delay/prevent disintegration of the MgO formulations tested under the experimental conditions. However, it does not establish the incidence, probability, or magnitude of esophageal obstruction in the broader dysphagic population. I recommend replacing categorical statements such as “constitutes a hazard” with other wordings."*

Response:

We thank the reviewer for this important conceptual clarification. We fully acknowledge that the phrase "constitutes a hazard embedded in current clinical systems" overstated the systemic and epidemiological implications beyond what our observational and *in vitro* data support.

We wish to clarify that we used the term "hazard" strictly in its epidemiological/safety-science sense—referring to an intrinsic physicochemical property of the drug–thickener combination capable of causing harm under stasis conditions, explicitly distinguished from "risk" (which requires quantitative probability and incidence estimates).

In accordance with the reviewer's recommendation, we have toned down and replaced categorical statements throughout the text. Specifically, "constitutes a hazard embedded in current clinical systems" has been replaced with *"represents a physicochemical hazard that may remain unrecognized in current clinical practice"* in the Abstract, Discussion, and Conclusion sections. We have also emphasized in the Limitations that prospective epidemiological studies are required to establish the incidence and clinical risk in the broader dysphagic population.

Comment 2:

*"2. The authors state that the findings demonstrate a “class effect.” However, only four MgO formulations were examined, and the study appears to be restricted to MgO tablets. Four formulations are useful for demonstrating reproducibility, but this does not necessarily establish a pharmaceutical “class effect.”"*

Response:

We agree with the reviewer's assessment. Testing four commercial MgO formulations demonstrates reproducibility across common Japanese products, but does not constitute formal proof of a universal pharmaceutical "class effect" across all magnesium oxide preparations or excipient variations.

We have removed the term "class effect" throughout the manuscript and replaced it with more precise descriptions, such as *"a consistent phenomenon observed across all tested formulations"* or *"a consistent behavior common to the tested MgO preparations."*

Comment 3:

*"3. The static model is appropriate as a screening approach, but the manuscript should discuss its limitations much more explicitly. The esophagus is not a static aqueous environment."*

Response:

We agree completely. The static immersion model was intentionally chosen to reproduce a low-shear stasis environment (such as that proximal to a malignant stricture), but it does not account for dynamic physiological factors present in an intact or functioning esophagus.

We have substantially expanded the discussion of this limitation in the Limitations section of the revised manuscript:

Comment 4:

*"4. The index case is the most clinically compelling component of the study, but additional information is necessary.*

*The manuscript should clarify:*

*• exact MgO dose and tablet strength;*

*• tablet manufacturer and formulation;*

*• time between administration and discovery;*

*• volume and viscosity/concentration of thickened water;*

*• xanthan gum product/brand;*

*• whether the tablet was administered intact or modified;"*

Response:

We thank the reviewer for this constructive suggestion. All requested details have been incorporated into the Case Presentation and Methods sections:

- Exact MgO dose and tablet strength: A single 330 mg tablet of magnesium oxide.
- Tablet manufacturer and formulation: Magnesium Oxide Tablets 330 mg "YOSHIDA" (Yoshida Pharmaceutical Co., Ltd., Tokyo, Japann; designated as F-O).
- Time between administration and discovery: Over 70 hours (the tablet was administered prior to referral and discovered during radiotherapy planning CT; the residue remained lodged on the day 3 setup CT).
- Volume and viscosity/concentration of thickened water: 100 mL of water thickened at 1.0–3.0% (w/v) (corresponding to Stages 1–3 [50–500 mPa·s] of the JSDR 2021 criteria, as identified through interviews with the 10 responsible ward nurses).
- Xanthan gum product/brand: Tsururinko Quickly (Clinico Co., Ltd., Tokyo, Japan; designated as TA-O).
- Administration state: The tablet was administered intact as a whole tablet (not crushed, split, or modified).

Comment 5:

*"5. The correct age should be verified throughout the manuscript, including the abstract, case description, tables, and supplementary information."*

Response:

We thank the reviewer for this careful observation. We have verified and standardized the patient's age as 81 years old consistently across the entire manuscript, including the Abstract, Introduction, Case Description, Tables, and Supporting Information files.

Comment 6:

*"6. The mechanism of MgO swelling/disintegration arrest should be investigated more deeply*

*The observation that tablet weight increased approximately 2.2-fold is interesting, but the mechanism deserves further discussion."*

Response:

We have expanded the mechanistic discussion in the Discussion section to explain the observed 2.2-fold weight increase, swelling, and disintegration arrest:

1. Water Sequestration: The xanthan gum polymer matrix binds and traps free water, drastically reducing water activity at the tablet surface and preventing the capillary hydration necessary to trigger tablet disintegrants.
2. Ionic Crosslinking ($Mg^{2+}$ – Xanthan Interaction): Divalent magnesium ions ($Mg^{2+}$) dissolving from the tablet surface bind to the pyruvate/glucuronate carboxyl groups on the side chains of xanthan gum molecules. This forms ionic crosslinks (junction zones), creating an insoluble, highly viscous gel envelope around the tablet that physically blocks further water ingress and arrests disintegration in a hydrated, swollen, and hardened state.

We have also noted in the Limitations that direct microscopic verification (e.g., via SEM) remains a subject for future structural investigations.

Comment 7:

*"7. The manuscript reports that 2.0–3.0% thickeners increased tablet weight approximately 2.2-fold and produced rigid masses. This is potentially important, but the relationship between viscosity and disintegration should be presented quantitatively."*

Response:

We have revised the Results and Discussion sections to provide quantitative details regarding the relationship between concentration/viscosity stages and disintegration outcomes:

- At 1.0% concentration (Stage 1, 50–150 mPa·s), tablets exhibited a *friable non-disintegration (FN)* state after 24 hours—retaining their gross shape but crumbling under minimal force.
- At 2.0% concentration (Stage 2, 150–300 mPa·s) and 3.0% concentration (Stage 3, 300–500 mPa·s), complete non-disintegration (*CN state*) was maintained even at 24 hours. The tablet weight increased by 2.2-fold to $0.91\pm0.02\text{ g}$ in 2.0% and $0.92\pm0.01\text{ g}$ in 3.0%, with no statistically significant difference between 2.0% and 3.0%, indicating that the hardening and water sequestration plateau above 2.0%.

Comment 8:

*"8. The rapid disintegration in SA-J is interesting, but the comparison may not be entirely equivalent to the thickened-water experiments.*

*The authors should explain:*

*• the composition of SA-J;*

*• water content;*

*• viscosity/rheological characteristics;*

*• pH;*

*• volume used;"*

Response:

We agree that the physical medium and administration method of SA-J inherently differ from the direct aqueous immersion of thickened water. We have explicitly detailed these parameters in the revised Materials and Methods section under "Medium preparation":

- Product and Composition: Okusuri Nometane (Ryukakusan Co., Ltd., Tokyo, Japan), an agar-based hydrogel matrix containing water, erythritol, buffering agents, and gelling agents (agar/pectin).
- Water Content: Approximately 85–90% bound water.
- Viscosity/Rheological Characteristics: Instrumental rheological measurements of SA-J were not performed, as appropriate rheological equipment was unavailable in our clinical setting — as acknowledged in the Limitations section. Furthermore, as SA-J is classified as a food product in Japan, no pharmaceutical package insert specifying viscosity parameters is available. However, SA-J is an agar-based hydrogel matrix that is physicochemically distinct from xanthan gum solutions: agar gels are thermoreversible and do not form ionic crosslinks with Mg²⁺ ions, which mechanistically explains the absence of disintegration inhibition observed in our experiments.
- pH: 6.0–6.5 (neutral to weakly acidic).
- Volume Used: One standard 18 g sachet, administered using the guideline-recommended sandwich method (encapsulating the tablet between two approximately 9 g portions of jelly), followed by immersion in distilled water.

In the Limitations section, we have also noted that while this experimental design was chosen to replicate real-world clinical administration protocols, the differences in media volume and physical matrix represent an inherent methodological distinction.
