# Supplementary material for: Magnesium oxide tablets form rigid masses in xanthan gum-based thickeners: A physicochemical hazard revealed by a clinical case
Source: PLoS One. 2026 Oct 29;21(10):e0361494. doi: 10.1371/journal.pone.0361494 (PMC13718963; doi:10.1371/journal.pone.0361494)
Supplement: Peer review history

# Peer review: Decision Letter 0

**DOI:** 10.1371/journal.pone.0361494.r001

**Authors:** Claudio Salomon

10 Aug 2026

Dear Dr. Makino,

Thank you for submitting your manuscript to PLOS ONE. After careful consideration, we feel that it has merit but does not fully meet PLOS ONE’s publication criteria as it currently stands. Therefore, we invite you to submit a revised version of the manuscript that addresses the points raised during the review process.

Please submit your revised manuscript by Oct 09 2026 11:59PM. If you will need more time than this to complete your revisions, please reply to this message or contact the journal office at plosone@plos.org. When you're ready to submit your revision, log on to https://www.editorialmanager.com/pone/ and select the 'Submissions Needing Revision' folder to locate your manuscript file.

- A letter that responds to each point raised by the academic editor and reviewer(s). You should upload this letter as a separate file labeled 'Response to Reviewers'.
- A marked-up copy of your manuscript that highlights changes made to the original version. You should upload this as a separate file labeled 'Revised Manuscript with Track Changes'.
- An unmarked version of your revised paper without tracked changes. You should upload this as a separate file labeled 'Manuscript'.

If you would like to make changes to your financial disclosure, please include your updated statement in your cover letter. Guidelines for resubmitting your figure files are available below the reviewer comments at the end of this letter.

If applicable, we recommend that you deposit your laboratory protocols in protocols.io to enhance the reproducibility of your results. Protocols.io assigns your protocol its own identifier (DOI) so that it can be cited independently in the future. For instructions see: https://journals.plos.org/plosone/s/submission-guidelines#loc-laboratory-protocols. Additionally, PLOS ONE offers an option for publishing peer-reviewed Lab Protocol articles, which describe protocols hosted on protocols.io. Read more information on sharing protocols at https://plos.org/protocols?utm_medium=editorial-email&utm_source=authorletters&utm_campaign=protocols.

As the corresponding author, your ORCID iD is verified in the submission system and will appear in the published article. PLOS supports the use of ORCID, and we encourage all coauthors to register for an ORCID iD and use it as well. Please encourage your coauthors to verify their ORCID iD within the submission system before final acceptance, as unverified ORCID iDs will not appear in the published article. *Only*  the individual author can complete the verification step; PLOS staff *cannot*  verify ORCID iDs on behalf of authors.

We look forward to receiving your revised manuscript.

Kind regards,

Claudio J. Salomon

Academic Editor

PLOS One

Journal Requirements:

When submitting your revision, we need you to address these additional requirements.

1. Please ensure that your manuscript meets PLOS ONE's style requirements, including those for file naming. The PLOS ONE style templates can be found at

https://journals.plos.org/plosone/s/file?id=wjVg/PLOSOne_formatting_sample_main_body.pdf and https://journals.plos.org/plosone/s/file?id=ba62/PLOSOne_formatting_sample_title_authors_affiliations.pdf

2. We note that Figure(s) 1, 2, 3, 4, 5, 6, S1 – S6, File S1, in your submission contain copyrighted images. All PLOS content is published under the Creative Commons Attribution License (CC BY 4.0), which means that the manuscript, images, and Supporting Information files will be freely available online, and any third party is permitted to access, download, copy, distribute, and use these materials in any way, even commercially, with proper attribution. For more information, see our copyright guidelines: http://journals.plos.org/plosone/s/licenses-and-copyright.

We require you to either (1) present written permission from the copyright holder to publish these figures specifically under the CC BY 4.0 license, or (2) remove the figures from your submission:

a. You may seek permission from the original copyright holder of Figure(s) 1, 2, 3, 4, 5, 6, S1 – S6, File S1, to publish the content specifically under the CC BY 4.0 license.

We recommend that you contact the original copyright holder with the Content Permission Form (http://journals.plos.org/plosone/s/file?id=7c09/content-permission-form.pdf) and the following text:

“I request permission for the open-access journal PLOS ONE to publish XXX under the Creative Commons Attribution License (CCAL) CC BY 4.0 (http://creativecommons.org/licenses/by/4.0/). Please be aware that this license allows unrestricted use and distribution, even commercially, by third parties. Please reply and provide explicit written permission to publish XXX under a CC BY license and complete the attached form.”

Please upload the completed Content Permission Form or other proof of granted permissions as an "Other" file with your submission.

In the figure caption of the copyrighted figure, please include the following text: “Reprinted from [ref] under a CC BY license, with permission from [name of publisher], original copyright [original copyright year].”

b. If you are unable to obtain permission from the original copyright holder to publish these figures under the CC BY 4.0 license or if the copyright holder’s requirements are incompatible with the CC BY 4.0 license, please either i) remove the figure or ii) supply a replacement figure that complies with the CC BY 4.0 license. Please check copyright information on all replacement figures and update the figure caption with source information. If applicable, please specify in the figure caption text when a figure is similar but not identical to the original image and is therefore for illustrative purposes only.

3. We notice that your supplementary figures are uploaded with the file type 'Figure'. Please amend the file type to 'Supporting Information'. Please ensure that each Supporting Information file has a legend listed in the manuscript after the references list.

4. If the reviewer comments include a recommendation to cite specific previously published works, please review and evaluate these publications to determine whether they are relevant and should be cited. There is no requirement to cite these works unless the editor has indicated otherwise.

[Note: HTML markup is below. Please do not edit.]

Reviewers' comments:

Reviewer's Responses to Questions

**Comments to the Author**

1. Is the manuscript technically sound, and do the data support the conclusions?

Reviewer #1: Partly

Reviewer #2: Yes

**********

2. Has the statistical analysis been performed appropriately and rigorously?

Reviewer #1: No

Reviewer #2: No

**********

3. Have the authors made all data underlying the findings in their manuscript fully available?

The PLOS Data policy

Reviewer #1: No

Reviewer #2: Yes

**********

4. Is the manuscript presented in an intelligible fashion and written in standard English?

Reviewer #1: No

Reviewer #2: Yes

**********

Reviewer #1: Dear SIR,

Entitle Magnesium oxide tablets form rigid masses in xanthan gum-based thickeners: A

physicochemical hazard revealed by a clinica"

reviewed need clarity explination , no images found , clearity method of adminstration protocol, no clinical data evidence and CT.

several grammatic and lingustic errors

Plagarism check require

Reviewer #2: The study contains a potentially important clinical and pharmaceutical observation and could be suitable for publication after substantial revision. The principal issue is not the experimental observation itself, which appears interesting and reproducible, but the extent to which the authors generalize the findings from a single clinical case and a static in vitro model to a broader population-level clinical hazard. Strengthening the methodological description, moderating the causal language, clarifying the mechanism, and providing a more rigorous economic analysis would considerably improve the manuscript.

1. The statement that xanthan gum-based thickened water “constitutes a hazard embedded in current clinical systems” is stronger than the evidence presented. The study demonstrates that xanthan gum thickeners can markedly delay/prevent disintegration of the MgO formulations tested under the experimental conditions. However, it does not establish the incidence, probability, or magnitude of esophageal obstruction in the broader dysphagic population. I recommend replacing categorical statements such as “constitutes a hazard” with other wordings.

2. The authors state that the findings demonstrate a “class effect.” However, only four MgO formulations were examined, and the study appears to be restricted to MgO tablets. Four formulations are useful for demonstrating reproducibility, but this does not necessarily establish a pharmaceutical “class effect.”

3. The static model is appropriate as a screening approach, but the manuscript should discuss its limitations much more explicitly. The esophagus is not a static aqueous environment.

4. The index case is the most clinically compelling component of the study, but additional information is necessary.

The manuscript should clarify:

• exact MgO dose and tablet strength;

• tablet manufacturer and formulation;

• time between administration and discovery;

• volume and viscosity/concentration of thickened water;

• xanthan gum product/brand;

• whether the tablet was administered intact or modified;

5. The correct age should be verified throughout the manuscript, including the abstract, case description, tables, and supplementary information.

6. The mechanism of MgO swelling/disintegration arrest should be investigated more deeply

The observation that tablet weight increased approximately 2.2-fold is interesting, but the mechanism deserves further discussion.

7. The manuscript reports that 2.0–3.0% thickeners increased tablet weight approximately 2.2-fold and produced rigid masses. This is potentially important, but the relationship between viscosity and disintegration should be presented quantitatively.

8. The rapid disintegration in SA-J is interesting, but the comparison may not be entirely equivalent to the thickened-water experiments.

The authors should explain:

• the composition of SA-J;

• water content;

• viscosity/rheological characteristics;

• pH;

• volume used;

**********

what does this mean?). If published, this will include your full peer review and any attached files.

If you choose “no”, your identity will remain anonymous but your review may still be made public.

**Do you want your identity to be public for this peer review?**  For information about this choice, including consent withdrawal, please see our Privacy Policy

Reviewer #1: No

Reviewer #2: No

**********

[NOTE: If reviewer comments were submitted as an attachment file, they will be attached to this email and accessible via the submission site. Please log into your account, locate the manuscript record, and check for the action link "View Attachments". If this link does not appear, there are no attachment files.]

To ensure your figures meet our technical requirements, please review our figure guidelines: https://journals.plos.org/plosone/s/figures

You may also use PLOS’s free figure tool, NAAS, to help you prepare publication quality figures: https://journals.plos.org/plosone/s/figures#loc-tools-for-figure-preparation.

NAAS will assess whether your figures meet our technical requirements by comparing each figure against our figure specifications.

# Author comment: Author response to Decision Letter 1

**DOI:** 10.1371/journal.pone.0361494.r002

20 Sep 2026

Response to Reviewer #1

Dear Reviewer #1,

We sincerely appreciate your review and comments on our manuscript (PONE-D-26-27019). We have carefully addressed each of your points and revised the manuscript accordingly. Point-by-point responses are provided below.

1. Clarity and Detailed Explanation

Reviewer Comment:

"reviewed need clarity explination"

Response:

We have substantially revised the Abstract, Introduction, and Discussion to more clearly explain the physicochemical mechanism of disintegration inhibition, the clinical relevance of esophageal impaction, and the overall study framework.

2. Availability of Figures, CT Images, and Clinical Data

Reviewer Comment:

"no images found/ no clinical data evidence and CT"

Response:

We apologize for the confusion regarding the availability of figures and CT images. In the initial submission, figure files were inadvertently uploaded within .docx wrappers rather than as standalone high-resolution image files, which caused rendering and display issues within the review system.

To fully resolve this issue:

Primary Figures and CT Images: All primary figures (Figs 1–6) have been converted to high-resolution TIFF files strictly following PLOS ONE specifications (Fig1.tif–Fig6.tif). These include the in vivo clinical CT findings of the impacted mass (Fig 2A and 2B) and the endoscopic confirmation (Fig 2C).

Supplementary Figures: All supplementary files — including the comparative ex vivo CT imaging (S4_Fig) and the recurrent case CT and clinical data (S5_Fig) — have been updated to standalone TIFF formats and reclassified under the "Supporting Information" category (S1_Fig.tif–S6_Fig.tif). Detailed captions for all Supporting Information items have been placed directly following the References section in the revised manuscript.

Clinical Evidence: Clinical data and CT evidence from the index case and the recurrent case serve as the clinical foundation of this study, complemented by systematic in vitro replication experiments.

3. Administration Protocol and In Vitro Methods

Reviewer Comment:

"clearity method of adminstration protocol"

Response:

We would like to clarify that no prospective drug administration to human subjects was performed for the experimental series. To ensure full transparency and reproducibility, the detailed protocol for the in vitro static immersion model (simulating esophageal stasis) and the clinical administration sandwich method used for swallowing aid jelly are documented in full detail in the Materials and Methods section.

4. English Grammar and Linguistic Quality

Reviewer Comment:

"several grammatic and lingustic errors"

Response:

The manuscript has undergone comprehensive professional English language editing by Editage (www.editage.com) prior to resubmission. A formal certificate of English editing is provided as a supplementary document. In accordance with PLOS ONE guidelines, we have declared the use of generative AI assistance (Gemini, Google LLC) for initial draft translation and syntactic structuring in the dedicated declaration section, after which all content was verified and refined by the authors and professional editors.

5. Plagiarism and Originality Check

Reviewer Comment:

"Plagarism check require"

Response:

We confirm that this manuscript represents original research and has been checked to ensure academic integrity. All figures, photographs, CT scans, schematics, and text are original works created by the authors specifically for this study. The third-party material previously included in File S1 has been completely removed to ensure full compliance with CC BY 4.0 licensing.

________________________________________

Response to Reviewer #2

Dear Reviewer #2,

We sincerely thank the reviewer for the thorough, constructive, and insightful evaluation of our manuscript (PONE-D-26-27019). The comments have helped us significantly improve the scientific precision, methodological transparency, and clinical contextualization of our work.

We have carefully addressed all points raised by the reviewer. Below are our point-by-point responses to the comments and details of the corresponding revisions made to the manuscript.

General Comments:

"The study contains a potentially important clinical and pharmaceutical observation and could be suitable for publication after substantial revision. The principal issue is not the experimental observation itself, which appears interesting and reproducible, but the extent to which the authors generalize the findings from a single clinical case and a static in vitro model to a broader population-level clinical hazard. Strengthening the methodological description, moderating the causal language, clarifying the mechanism, and providing a more rigorous economic analysis would considerably improve the manuscript."

Response:

We are grateful for these encouraging and constructive overarching remarks. We agree that our initial framing over-generalized the clinical findings beyond the immediate empirical scope. In the revised manuscript, we have moderated our causal language and avoided overgeneralization throughout, clarified that our study characterizes an intrinsic physicochemical hazard under static stasis conditions rather than a quantified population risk, expanded the methodological and mechanistic details, and presented the retail market economic survey with appropriate caveats.

Comment 1:

"1. The statement that xanthan gum-based thickened water “constitutes a hazard embedded in current clinical systems” is stronger than the evidence presented. The study demonstrates that xanthan gum thickeners can markedly delay/prevent disintegration of the MgO formulations tested under the experimental conditions. However, it does not establish the incidence, probability, or magnitude of esophageal obstruction in the broader dysphagic population. I recommend replacing categorical statements such as “constitutes a hazard” with other wordings."

Response:

We thank the reviewer for this important conceptual clarification. We fully acknowledge that the phrase "constitutes a hazard embedded in current clinical systems" overstated the systemic and epidemiological implications beyond what our observational and in vitro data support.

We wish to clarify that we used the term "hazard" strictly in its epidemiological/safety-science sense—referring to an intrinsic physicochemical property of the drug–thickener combination capable of causing harm under stasis conditions, explicitly distinguished from "risk" (which requires quantitative probability and incidence estimates).

In accordance with the reviewer's recommendation, we have toned down and replaced categorical statements throughout the text. Specifically, "constitutes a hazard embedded in current clinical systems" has been replaced with "represents a physicochemical hazard that may remain unrecognized in current clinical practice" in the Abstract, Discussion, and Conclusion sections. We have also emphasized in the Limitations that prospective epidemiological studies are required to establish the incidence and clinical risk in the broader dysphagic population.

Comment 2:

"2. The authors state that the findings demonstrate a “class effect.” However, only four MgO formulations were examined, and the study appears to be restricted to MgO tablets. Four formulations are useful for demonstrating reproducibility, but this does not necessarily establish a pharmaceutical “class effect.”"

Response:

We agree with the reviewer's assessment. Testing four commercial MgO formulations demonstrates reproducibility across common Japanese products, but does not constitute formal proof of a universal pharmaceutical "class effect" across all magnesium oxide preparations or excipient variations.

We have removed the term "class effect" throughout the manuscript and replaced it with more precise descriptions, such as "a consistent phenomenon observed across all tested formulations" or "a consistent behavior common to the tested MgO preparations."

Comment 3:

"3. The static model is appropriate as a screening approach, but the manuscript should discuss its limitations much more explicitly. The esophagus is not a static aqueous environment."

Response:

We agree completely. The static immersion model was intentionally chosen to reproduce a low-shear stasis environment (such as that proximal to a malignant stricture), but it does not account for dynamic physiological factors present in an intact or functioning esophagus.

We have substantially expanded the discussion of this limitation in the Limitations section of the revised manuscript:

Comment 4:

"4. The index case is the most clinically compelling component of the study, but additional information is necessary.

The manuscript should clarify:

• exact MgO dose and tablet strength;

• tablet manufacturer and formulation;

• time between administration and discovery;

• volume and viscosity/concentration of thickened water;

• xanthan gum product/brand;

• whether the tablet was administered intact or modified;"

Response:

We thank the reviewer for this constructive suggestion. All requested details have been incorporated into the Case Presentation and Methods sections:

Exact MgO dose and tablet strength: A single 330 mg tablet of magnesium oxide.

Tablet manufacturer and formulation: Magnesium Oxide Tablets 330 mg "YOSHIDA" (Yoshida Pharmaceutical Co., Ltd., Tokyo, Japann; designated as F-O).

Time between administration and discovery: Over 70 hours (the tablet was administered prior to referral and discovered during radiotherapy planning CT; the residue remained lodged on the day 3 setup CT).

Volume and viscosity/concentration of thickened water: 100 mL of water thickened at 1.0–3.0% (w/v) (corresponding to Stages 1–3 [50–500 mPa·s] of the JSDR 2021 criteria, as identified through interviews with the 10 responsible ward nurses).

Xanthan gum product/brand: Tsururinko Quickly (Clinico Co., Ltd., Tokyo, Japan; designated as TA-O).

Administration state: The tablet was administered intact as a whole tablet (not crushed, split, or modified).

Comment 5:

"5. The correct age should be verified throughout the manuscript, including the abstract, case description, tables, and supplementary information."

Response:

We thank the reviewer for this careful observation. We have verified and standardized the patient's age as 81 years old consistently across the entire manuscript, including the Abstract, Introduction, Case Description, Tables, and Supporting Information files.

Comment 6:

"6. The mechanism of MgO swelling/disintegration arrest should be investigated more deeply

The observation that tablet weight increased approximately 2.2-fold is interesting, but the mechanism deserves further discussion."

Response:

We have expanded the mechanistic discussion in the Discussion section to explain the observed 2.2-fold weight increase, swelling, and disintegration arrest:

Water Sequestration: The xanthan gum polymer matrix binds and traps free water, drastically reducing water activity at the tablet surface and preventing the capillary hydration necessary to trigger tablet disintegrants.

Ionic Crosslinking (Mg^(2+) – Xanthan Interaction): Divalent magnesium ions (Mg^(2+)) dissolving from the tablet surface bind to the pyruvate/glucuronate carboxyl groups on the side chains of xanthan gum molecules. This forms ionic crosslinks (junction zones), creating an insoluble, highly viscous gel envelope around the tablet that physically blocks further water ingress and arrests disintegration in a hydrated, swollen, and hardened state.

We have also noted in the Limitations that direct microscopic verification (e.g., via SEM) remains a subject for future structural investigations.

Comment 7:

"7. The manuscript reports that 2.0–3.0% thickeners increased tablet weight approximately 2.2-fold and produced rigid masses. This is potentially important, but the relationship between viscosity and disintegration should be presented quantitatively."

Response:

We have revised the Results and Discussion sections to provide quantitative details regarding the relationship between concentration/viscosity stages and disintegration outcomes:

At 1.0% concentration (Stage 1, 50–150 mPa·s), tablets exhibited a friable non-disintegration (FN) state after 24 hours—retaining their gross shape but crumbling under minimal force.

At 2.0% concentration (Stage 2, 150–300 mPa·s) and 3.0% concentration (Stage 3, 300–500 mPa·s), complete non-disintegration (CN state) was maintained even at 24 hours. The tablet weight increased by 2.2-fold to 0.91±0.02" g" in 2.0% and 0.92±0.01" g" in 3.0%, with no statistically significant difference between 2.0% and 3.0%, indicating that the hardening and water sequestration plateau above 2.0%.

Comment 8:

"8. The rapid disintegration in SA-J is interesting, but the comparison may not be entirely equivalent to the thickened-water experiments.

The authors should explain:

• the composition of SA-J;

• water content;

• viscosity/rheological characteristics;

• pH;

• volume used;"

Response:

We agree that the physical medium and administration method of SA-J inherently differ from the direct aqueous immersion of thickened water. We have explicitly detailed these parameters in the revised Materials and Methods section under "Medium preparation":

Product and Composition: Okusuri Nometane (Ryukakusan Co., Ltd., Tokyo, Japan), an agar-based hydrogel matrix containing water, erythritol, buffering agents, and gelling agents (agar/pectin).

Water Content: Approximately 85–90% bound water.

Viscosity/Rheological Characteristics: Instrumental rheological measurements of SA-J were not performed, as appropriate rheological equipment was unavailable in our clinical setting — as acknowledged in the Limitations section. Furthermore, as SA-J is classified as a food product in Japan, no pharmaceutical package insert specifying viscosity parameters is available. However, SA-J is an agar-based hydrogel matrix that is physicochemically distinct from xanthan gum solutions: agar gels are thermoreversible and do not form ionic crosslinks with Mg²⁺ ions, which mechanistically explains the absence of disintegration inhibition observed in our experiments.

pH: 6.0–6.5 (neutral to weakly acidic).

Volume Used: One standard 18 g sachet, administered using the guideline-recommended sandwich method (encapsulating the tablet between two approximately 9 g portions of jelly), followed by immersion in distilled water.

In the Limitations section, we have also noted that while this experimental design was chosen to replicate real-world clinical administration protocols, the differences in media volume and physical matrix represent an inherent methodological distinction.

Attachment Submitted filename: Response_to_Reviewers_PONE-D-26-27019.docx

# Peer review: Decision Letter 1

**DOI:** 10.1371/journal.pone.0361494.r003

**Authors:** Claudio Salomon

12 Oct 2026

Magnesium oxide tablets form rigid masses in xanthan gum-based thickeners: A physicochemical hazard revealed by a clinical case

PONE-D-26-27019R1

Dear Dr. Makino,

We’re pleased to inform you that your manuscript has been judged scientifically suitable for publication and will be formally accepted for publication once it meets all outstanding technical requirements.

Within one week, you’ll receive an e-mail detailing the required amendments. When these have been addressed, you’ll receive a formal acceptance letter and your manuscript will be scheduled for publication.

An invoice will be generated when your article is formally accepted. Please note, if your institution has a publishing partnership with PLOS and your article meets the relevant criteria, all or part of your publication costs will be covered. Please make sure your user information is up-to-date by logging into Editorial Manager at Editorial Manager® and clicking the ‘Update My Information' link at the top of the page. For questions related to billing, please contact billing support.

If your institution or institutions have a press office, please notify them about your upcoming paper to help maximize its impact. If they’ll be preparing press materials, please inform our press team as soon as possible -- no later than 48 hours after receiving the formal acceptance. Your manuscript will remain under strict press embargo until 2 pm Eastern Time on the date of publication. For more information, please contact onepress@plos.org.

Kind regards,

Claudio J. Salomon

Academic Editor

PLOS One

Additional Editor Comments (optional):

Reviewers' comments:

Reviewer's Responses to Questions

**Comments to the Author**

Reviewer #2: All comments have been addressed

**********

2. Is the manuscript technically sound, and do the data support the conclusions?

Reviewer #2: Yes

**********

3. Has the statistical analysis been performed appropriately and rigorously?

Reviewer #2: Yes

**********

4. Have the authors made all data underlying the findings in their manuscript fully available?

The PLOS Data policy

Reviewer #2: Yes

**********

5. Is the manuscript presented in an intelligible fashion and written in standard English?

Reviewer #2: Yes

**********

Reviewer #2: (No Response)

**********

what does this mean?). If published, this will include your full peer review and any attached files.

If you choose “no”, your identity will remain anonymous but your review may still be made public.

**Do you want your identity to be public for this peer review?**  For information about this choice, including consent withdrawal, please see our Privacy Policy

Reviewer #2: No

**********

# Editor report: Acceptance letter

**DOI:** 10.1371/journal.pone.0361494.r004

**Authors:** Claudio Salomon

PONE-D-26-27019R1

PLOS One

Dear Dr. Makino,

I'm pleased to inform you that your manuscript has been deemed suitable for publication in PLOS One. Congratulations! Your manuscript is now being handed over to our production team.

At this stage, our production department will prepare your paper for publication. This includes ensuring the following:

* All references, tables, and figures are properly cited

* All relevant supporting information is included in the manuscript submission,

* There are no issues that prevent the paper from being properly typeset

You will receive further instructions from the production team, including instructions on how to review your proof when it is ready. Please keep in mind that we are working through a large volume of accepted articles, so please give us a few days to review your paper and let you know the next and final steps.

Lastly, if your institution or institutions have a press office, please let them know about your upcoming paper now to help maximize its impact. If they'll be preparing press materials, please inform our press team within the next 48 hours. Your manuscript will remain under strict press embargo until 2 pm Eastern Time on the date of publication. For more information, please contact onepress@plos.org.

You will receive an invoice from PLOS for your publication fee after your manuscript has reached the completed accept phase. If you receive an email requesting payment before acceptance or for any other service, this may be a phishing scheme. Learn how to identify phishing emails and protect your accounts at https://explore.plos.org/phishing.

If we can help with anything else, please email us at customercare@plos.org.

Thank you for submitting your work to PLOS One and supporting open access.

Kind regards,

PLOS One Editorial Office Staff

on behalf of

Dr. Claudio J. Salomon

Academic Editor

PLOS One
